# Brainstem Auditory Evoked Potentials in infants aged 1 to 24 months during a hearing health care service

**DOI:** 10.6061/clinics/2020/e1579

**Published:** 2020-10-21

**Authors:** Cintia Gonçalves de Lima Bellia, Haraldo Artmann Junior, Jair Mendes Marques, Débora Lüders, Cláudia Giglio de Oliveira Gonçalves

**Affiliations:** Universidade Tuiuti do Parana, Curitiba, PR, BR

**Keywords:** Electrophysiology, Hearing, Brainstem Auditory Evoked Potential, Infants

## Abstract

**OBJECTIVES:**

Assessing infants’ hearing is of utmost importance, as hearing at this phase is required for the development of oral language. Through hearing, human beings are capable of developing communication. The Brainstem Auditory Evoked Potentials are an indispensable test to diagnose deafness in infants. This study aimed to analyze the results of the Brainstem Auditory Evoked Potentials in children with risk factors for hearing loss.

**METHODS:**

This cross-sectional study analyzed the Brainstem Auditory Evoked Potentials in 123 infants aged 1 to 24 months at a hearing health care service. The Vivosonic Integrity V500 equipment, which enabled the child to be awake while the testing was carried out, was used in this study. The data were compared by gestational age and sex, according to the standards suggested in the equipment handbook.

**RESULTS:**

A significant difference was verified for age ranges 4 to 6 months, 13 to 15 months (waves I and V), and 7 to 9 months (wave V). The lower values in absolute wave latencies were comparable to data from the equipment handbook, justifying the need for standardization of the screening process.

**CONCLUSION:**

There are some differences between the standards in the equipment handbook and those observed in our study. These results will serve as a reference for the standardization of the equipment used in the hearing health care service.

## INTRODUCTION

The hearing assessment in infants is of utmost importance, as hearing in this phase is required for the normal development of oral language and cognitive functions. Hearing allows human beings to identify, locate, and develop sensory experiences; this contributes not only to the maturation of the hearing system, but also the cognitive and emotional functions. It is also essential for acquisition and development of the oral and written language.

Several organizations (1) and committees have established guidelines to implement Newborn Hearing Screening (NHS). In Brazil, the Universal Newborn Hearing Screening (UNHS) became mandatory under Federal Law 12,303 and began to be implemented in August 2010, following the model of developed countries (2).

Newborn Hearing Screening (NHS) aims for the early detection of cochlear hearing loss greater than 35 dB HL and may also comprise retrocochlear hearing loss among neonates and infants. Newborn Hearing Screening (NHS) is carried out by means of physiological and electrophysiological hearing measures used to assess the peripheral and central auditory pathways (3). In Universal Newborn Hearing Screening (UNHS), there are several actions that need to be performed, including screening, monitoring and performing follow-up assessments for hearing and language development, diagnosis, and rehabilitation, ensuring comprehensive care in childhood, since children diagnosed with hearing loss before 6 months of age have a greater chance of having normal speech development, learning skills, and better quality of life (4).

The Brainstem Auditory Evoked Potentials (BAEP) is an important tool for the diagnosis of hearing impairment in neonates and infants, since it assesses the integrity of the auditory pathways and their maturation in relation to the infants’ age on the day of the test. A previous study (5) reported that when a neonate fails the hearing screening, the most important steps to take include verifying the integrity of the auditory pathway, identifying probable retrocochlear hearing loss, determining minimum levels of responses or thresholds at different frequencies and, if applicable, identifying the type of hearing loss.

Brainstem Auditory Evoked Potentials (BAEP), permits assessment of the integrity of the auditory pathway to the brainstem, and identifies electrophysiological threshold in neonates, allowing for the diagnosis of child cochlear or retrocochlear hearing loss (6).

The Auditory Evoked Potentials (AEP) are electrophysiological responses to an acoustic stimulus and provide an objective measure of the integrity of the hearing system as a whole (3,7). The AEPs consist of a recording of the electrical activity in the auditory system, from the inner ear to the brain cortex, as a response to an acoustic stimulus, and provide information on the maturation of the brainstem auditory pathway (8). They can be detected by means of electrodes placed in strategic sites on the patient’s skin and recorded using the proper equipment, connected with a computer (5), with different classifications depending on the recording time.

Studies (5,6,9,10) report the age effect on the latencies of Brainstem Auditory Evoked Potentials (BAEP) waves, having detected important changes in the maturation process of the brainstem auditory pathways, which occur in full-term and premature neonates. Such changes continue throughout the first year of life, and by the end of the second year, the latencies of neonates are equal to those of adults. Previous studies (3,6,11) report that AEPs of adults and children differ. Only infants that are at least 18 to 24 months of age have responses that pair with those of adults (6); thus, morphological wave features vary based on age. Some studies (6,11) report that waves I, III, and V can be analyzed from the 30th to the 32nd week of gestational age. Wave latency I can be close to normal at 3 months of age (6,11), and waves III and V decrease during the first year of life, in which full-term infants between 18 and 24 months display similar values to those observed in adults. In case of premature neonates, age must be corrected based on the gestational time at the time of testing. Regarding the types of probable responses, there are regulation parameters for each equipment, as well as references of regulation values from different services that can be found in the literature (12,13).

Based on literature and equipment features, the expected normality standards of the Brainstem Auditory Evoked Potentials (BAEP) devices for each subject’s age can be found in their handbooks and software. These data, provided by the handbooks, may not reflect normality of a different population on which the equipment is used. Thus, literature recommends a biological calibration of the equipment, according to the screened population (5,8,12).

This study aims to analyze the results of Brainstem Auditory Evoked Potentials (BAEP) in children with risk factors for hearing loss.

## MATERIAL AND METHODS

This is a cross-sectional study, which analyzed the Brainstem Auditory Evoked Potentials (BAEP) results in 123 infants between 1 and 24 months of age at an accredited clinic of the Brazilian Unified Health System (Public Service-High Complexity Service in Hearing Health care). The children were referred to the hearing health care service to undergo hearing diagnosis when they presented risk factors for hearing loss or failed the Newborn Hearing Screening (NHS).

The study was approved by the Ethics Research Board under number 105.576. The free informed consent form was signed by parents/legal guardians before initiating the procedures.

The inclusion factors for the research were as follows: children presenting a hearing assessment result within the normal standards based on Brainstem Auditory Evoked Potentials (BAEP), considering the electrophysiological threshold at 25 dBHL, tested with at least two wave markings, corresponding to waves I, III and V, in both ears, and disclosure of all anamnesis data. Testing was carried out by audiologists from the service, with the same standards as those used to carry out Brainstem Auditory Evoked Potentials (BAEP), result interpretation, and diagnostic report. Out of the 123 infants assessed, 57 (46.34%) were female and 66 (53.65%) were male.

VIVOSONIC, Integrity Sistem^™^ V500 (14) equipment was used. By using the Vivosonic equipment, the child could be screened when they were either awake (15,16) or asleep without sedation; they could even be screened if they were playing quietly, but not if they were restless or crying.

The protocol used to carry out the testing was as follows (14): the children’´s skin was cleaned with alcohol and abrasive/conducting paste; the recording electrodes (Neuroline^Tm^ 720-00-S) were placed as recommended by the handbook, on both mastoids (negatives - left M1 and right M2), high forehead (positive - Fz), and low forehead (ground - Fpz). The impedance between the electrodes was less than 5 KOhms; broadband rarefaction click stimuli were presented by insertion phones (ER 3) at an initial intensity of 80 dBHL with a repetition rate of 27.5 clicks/second and an average of 2.000 stimuli. The sweep duration was 12 ms, and the equipment used the Kalman weighted algorithm for signal processing. Duplicate recordings were made to examine reproducibility. The acoustic stimulus was later presented decreasing intensities, every 20 dBHL, up to the limit of 25 dBHL, a value considered normal.

For data analysis, such as absolute latencies of waves I, III and V, the interpeak intervals I-III, III-V and I-V were considered at an intensity of 80 dBHL and were also analyzed as interaural differences of wave V. Consider as age groups in months. These data were compared to equipment standards. Differences between the right and left ears and sex were analyzed. Preterm infants, born at less than 37 weeks, had their ages corrected using the Capurro method (17).

Software Statística 13.3 and Mat Lab 6.0 and statistical procedures, such as the odds ratio test, Student’s t-test, and analyses of central tendency (means, standard deviation, medians) of the findings were used. In addition, data were compared by gestational age and sex, based on the standards suggested in the equipment handbook. The adopted level of significance was 0.05 (5%).

## RESULTS

**Figure 1:**
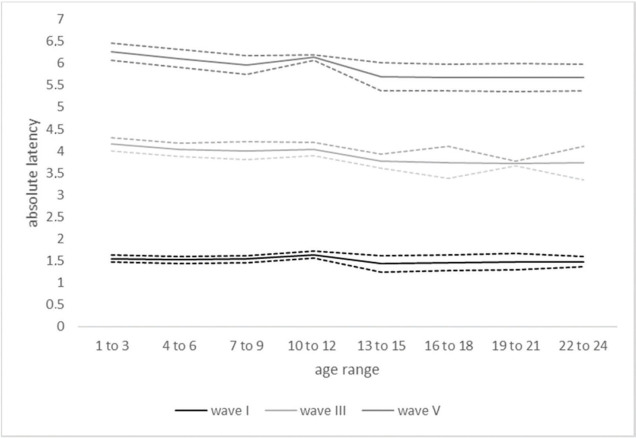
Evolution of absolute latencies of waves I, III, and V (Mean Results and Standard Deviations) by age range (N=123).

Table 1 shows the values found for absolute latencies and interpeak intervals by age range in months, and Figure 1 shows the evolution of absolute latencies, mean results, and standard deviations of waves I, III and V by age range in months.

In Table 1, an unsteady decrease in the absolute latencies and interpeak intervals was observed, correlated to the increase in chronological age.

In Table 2, a statistically significant difference was observed at age 1 to 3 months in interpeak intervals I-III, III-V, and I-V, which was greater among male infants. In the age range between 7 and 9 months, a statistically significant difference occurred in absolute latency of wave III, which was also greater among male infants.

Table 3 shows the comparison of the main result of BAEP (Brainstem Auditory Evoked Potentials) by gender and age range for the left ear.

There was a statistically significant difference in interpeak intervals I-III, III-V, and I-V, which was greater among male infants between ages 1 and 3 months. In the age range between 4 and 6 months, a statistically significant difference was observed in absolute latencies of waves III and V, and interpeak intervals I-II and I-V, which was also greater among male infants. In addition, in the age range from 7 to 9 months, a statistically significant difference was observed in the absolute latency of wave III, and interpeak intervals I-III, which was also greater among male infants.

Table 4 shows the comparisons between absolute latencies of waves observed in this study and the latencies based on the equipment standards.

We compared the sample means to the standard mean (equipment mean) using the Student’s t-test at significance level of 0.05 (5%) and verified a significant difference for age ranges from 4 to 6 months, 13 to 15 months at absolute latencies of waves I and V, and for the age range from 7 to 9 months at wave V. The test was not applied to ages ranging from 10 to 12 months, 15 to 18 months, and 18 to 21 months, since the samples for these age ranges were very small.

## DISCUSSION

The population in the study evidenced normal hearing at the time of data collection, identified by the behavioral assessment and Brainstem Auditory Evoked Potentials (BAEP), with the presence of absolute latencies of waves I, III, V, interpeak intervals I-III, III-V, and I-V, hearing thresholds up to 25 Dbhl.

Notably, normative data based on the literature, is used in BAEP equipment for specific populations for correct identification of the hearing loss, early diagnosis, and early initiation of a rehabilitation program when applicable (5,6,12).

Regarding Brainstem Auditory Evoked Potentials (BAEP) standardization, in this study (Table 1), a decrease in latency time based on age was observed at waves III and V, and interpeak intervals I-III, I-V, and III-V. This finding corroborates previous literature concerning the maturation of the auditory pathways, with a consequent decrease in the absolute latencies at waves I, III, and V in infants from 0 to 18 months of age. The hearing system matures in two distinct stages: the first occurs during pregnancy, ending around the 6 month mark, when the peripheral auditory pathways are fully developed; and the second, from birth to 18 months of age, when the maturation of the central nervous system, auditory pathways, and brainstem is verified (6,11). At this stage of the maturation process, authors believe that compared to older infants and adults, the increase in the latencies of waves occurs because of the slower electrical information in the group of younger infants. This electrical “lagging” that is verified in younger infants occurs because their auditory pathways are still developing, which includes the progressive increase in the myelination and axial diameter of nerves, enhancement of the neural synchrony, establishment of new effective connections, and greater synaptic efficiency (13). As age increases, absolute latencies of waves decrease. Authors state that this process of maturation occurs until 18 to 20 months of age, when latency values pair with those of adults (3,11,18,19).

Instead of the expected latency decrease because of the maturation process, a significant increase in absolute latency of wave I was observed as infants grew older. Some factors may have influenced this finding, referring to the profile of the babies in the present study, because at the ages of 6 to 24 months of age, babies do not sleep easily for the proper examination, which may have influenced recording of wave I. However, a Brazilian study (20), which analyzed probable differences in absolute latencies and interpeak intervals between the assessed situations, asleep and awake, with the same equipment used in the current study (Integrity V500), did not find statistically significant differences (20). Another possibility for the increase in the absolute latency of wave I could be the features of the equipment, recording wave I slightly above the predicted standard. Other authors (5) observed that small changes in wave I, with lower or higher values compared to the standard values, are acceptable when the expected standard deviation is maintained. Alternatively, an increase in the absolute latency of wave I could be either because of the subjectivity of the wave recording, performed by the professional, not the equipment (21), or the sampling size in this study. It is possible that different data could be obtained in a larger sample.

Studies show a symmetry between the ears; therefore, results found in the right ear can be correlated to those in the left ear (13,19,22). This similarity is expected because of the potential of the auditory nerve, which specifically reflects the ipsilateral responses of waves I and II. As for waves III, IV, and V, contralateral responses are verified, and it is possible that they are greater in number than the ipsilateral responses, thus presenting a more symmetrical response between the ears than at waves I and III. In the current study, as shown in Tables 2 and 3, statistically significant differences were observed in interpeak intervals I-III, III-V, and I-V at ages between 1 and 3 months, which was greater among male infants. At ages 4 to 6 months, statistically significant differences were observed at absolute latencies of waves III and V, and latency interpeak intervals I-II and I-V, which was also greater among male infants.

Additionally, at ages 7 to 9 months, a statistically significant difference was verified in the absolute latency of wave III and interpeak intervals I-III, which was also greater among male infants. Based on previous studies (12,23,24), these results may occur because of the differences in the diameter of the auditory nerve in male infants compared to female infants.

A comparison between our study data and standard equipment data (Table 4) revealed that our findings of the absolute latencies of waves were lower than of those based on equipment standards. A possibility that justifies this situation may be the unique features of the infants in this study, which may differ from those of the population used by the manufacturer to standardize the equipment, since most studies were carried out over 10 years ago. Thus, the standardization of Brainstem Auditory Evoked Potentials (BAEP) results is recommended for services in which Brainstem Auditory Evoked Potentials (BAEP) will be carried out (5,25).

Limitations of this study include use of a sample with infants at risk of hearing loss.

However, all infants underwent electrophysiological threshold assessment and those with hearing loss were excluded. In addition, gestational age was also corrected for premature infants, avoiding Brainstem Auditory Evoked Potentials (BAEP) distortions because of the immaturity of the auditory pathways. Some studies observed differences between Brainstem Auditory Evoked Potentials (BAEP) responses from neonates born preterm and at term, showing that responses generated between peripheral and central auditory pathways were influenced by the maturation process and gestational age (3,7,9,14). The current study did not consider risk factors for hearing loss as a variable. Some authors considered that Brainstem Auditory Evoked Potentials (BAEP) responses might be influenced by complications related to the risk factors for hearing loss, delaying the process of auditory pathway maturation. Studies showed to the effect of low Apgar scores (10), low birth weight (3,7), and the need of ICU care (3,7,10) on Brainstem Auditory Evoked Potentials (BAEP) responses. Some authors (3) recommend analysis of interference by the risk factors of hearing loss on Brainstem Auditory Evoked Potentials (BAEP) waves when the maturation process of children’s auditory pathways is complete.

## CONCLUSION

Lower values were found in the absolute latencies of waves I and V compared to the standard equipment data and non-reported values in the equipment handbook were found in the absolute latencies of wave III, justifying the need for standardization of the service responsible for performing the assessment.

By comparing the latencies of waves found in the study to those reported for the equipment, differences were verified in waves I and V at ages 4 to 6 months and 13 to 15 months, which was also verified in wave V at ages ranging from 7 to 9 months.

The values ??found in this study, for waves I, III and V from one to three months will serve as a basis for the analysis of the results of the babies attended at the studied hearing health service.

The results will serve as a reference for the standardization of equipment used in the hearing healthcare service, specifically for neonates with risk factors for hearing loss. Therefore, biological calibration is emphasized in the Brainstem Auditory Evoked Potentials (BAEP) test.

However, further research with larger samples is needed to confirm our study findings.

## AUTHOR CONTRIBUTIONS

Bellia CGL contributed to the project and production of the article: research design, conceptualization, data curation, formal analysis, investigation, methodology, and writing. Junior HA contributed with data curation, investigation, methodology, visualization, and writing. Marques JM contributed with methodology, validation, and formal analysis. Lüders D contributed with methodology, formal analysis, writing-review, and editing. Gonçalves CGO contributed with project administration, conceptualization, formal analysis, supervision, methodology, writing, and review.

## Figures and Tables

**Table 1:** Brainstem Auditory Evoked Potentials Mean Results and Standard Deviations (SD) by age range (n=123).

Age range (months)	Waves			Interpeak intervals		
	I	III	V	I-III	III-V	I-V
1-3 (n=34)	1.55	4.16	6.26	2.62	2.09	4.74
SD	0.08	0.15	0.19	0.15	0.16	0.30
4-6 (n=49)	1.52	4.03	6.11	2.52	2.05	4.59
SD	0.08	0.15	0.20	0.15	0.21	0.21
7-9 (n=16)	1.54	4.01	5.96	2.48	1.95	4.43
SD	0.08	0.20	0.22	0.20	0.17	0.23
10-12 (n=2)	1.64	4.04	6.13	2.40	2.09	4.50
SD	0.08	0.15	0.07	0.19	0.19	0.02
13-15 (n=10)	1.43	3.77	5.70	2.33	1.97	4.30
SD	0.18	0.16	0.32	0.26	0.16	0.50
16-18 (n=3)	1.46	3.74	5.67	2.29	1.94	4.23
SD	0.18	0.37	0.30	0.39	0.25	0.30
19-21 (n=3)	1.48	3.72	5.67	2.27	1.94	4.17
SD	0.19	0.05	0.32	0.09	0.21	0.18
22-24 (n=6)	1.48	3.73	5.68	2.26	1.90	4.20
SD	0.12	0.38	0.30	0.20	0.34	0.37

**Table 2:** Comparison of the Brainstem Auditory Evoked Potentials Results (Mean) by sex and age range for the right ear (n=123).

Male							Female						
Age range (months)	Absolute wave latencies			Interpeak intervals			Age range (months)	Absolute wave latencies			Interpeak intervals		
	I	III	V	I-III	III-V	I-V		I	III	V	I-III	III-V	I-V
1-3 (n=19)	1.54	4.17	6.27	2.63*	2.11*	4.73*	1-3 (n=15)	1.57	4.15	6.28	2.58*	2.09*	4.71*
4-6 (n=23)	1.52	4.10	6.15	2.57	2.02	4.63	4-6 (n=26)	1.53	4.03	6.09	2.51	2.05	4.55
7-9 (n=10)	1.56	4.10*	6.04	2.54	1.94	4.49	7-9 (n=6)	1.52	3.86*	5.83	2.35	1.96	4.31
10-12 (n=1)	NA	NA	NA	NA	NA	NA	10-12 (n=1)	NA	NA	NA	NA	NA	NA
13-15 (n=4)	1.46	3.70	5.63	2.29	1.90	4.20	13-15 (n=6)	1.52	3.90	5.81	2.44	2.06	4.30
16-18 (n=1)	NA	NA	NA	NA	NA	NA	16-18 (n=2)	1.50	3.77	5.42	2.26	1.66	3.91
19-21 (n=2)	1.68	3.91	5.86	2.45	1.75	4.18	19-21 (n=1)	NA	NA	NA	NA	NA	NA
22-24 (n=3)	1.60	3.90	4.40	2.26	1.94	4.40	22-24 (n=3)	1.41	3.71	5.55	2.29	1.84	4.13

Note: *Student’s t-test, significance level of 0.05 (5%); NA=not applicable (insufficient number of subjects). Values in milliseconds.

**Table 3:** Comparison of the Brainstem Auditory Evoked Potential Results (Mean) by sex and age range for the left ear (n=123).

Male							Female						
Age range (months)	Absolute latencies of waves			Interpeak intervals			Age range (months)	Absolute latencies of waves			Interpeak intervals		
	I	III	V	I-III	III-V	I-V		I	III	V	I-III	III-V	I-V
1-3 (n=19)	1.55	4.17	6.26	2.63*	2.12*	4.81*	1-3 (n=15)	1.53	4.15	6.24	2.61*	2.02*	4.71*
4-6 (n=23)	1.52	4.09*	6.18*	2.56*	2.08	4.65*	4-6 (n=26)	1.51	3.98*	6.04*	2.47*	2.06	4.53*
7-9 (n=10)	1.50	4.09*	6.04	2.59*	1.94	4.54	7-9 (n=6)	1.54	3.87*	5.85	2.32*	1.98	4.31
10-12 (n=1)	NA	NA	NA	NA	NA	NA	10-12 (n=1)	NA	NA	NA	NA	NA	NA
13-15 (n=6)	1.44	3.73	5.72	2.31	1.97	4.27	13-15 (n=4)	1.41	3.83	5.71	2.47	1.88	4.62
16-18 (n=1)	NA	NA	NA	NA	NA	NA	16-18 (n=2)	1.62	3.77	5.48	2.14	1.71	3.88
19-21 (n=2)	1.61	3.87	5.80	2.45	1.64	4.19	19-21 (n=1)	NA	NA	NA	NA	NA	NA
22-24 (n=3)	1.58	3.76	5.99	2.23	2.23	4.4	22-23 (n=3)	1.48	3.75	5.60	2.24	1.84	4.10

* Note: Student’s t-test at a significance level of 0.05 (5%); NA=not applicable (insufficient number of subjects).

**Table 4:** Absolute wave latencies by age group from 1 to 24 months (comparison between results and equipment standards).

Age range (months)	Absolute latencies waves I and V - Study		Absolute latencies waves I and V -Equipment		*p*	*p*
	I	V	I	V	I	V
1-3 (n=34)	1.55	6.27	-	-		
SD	0.08	0.20				
4-6 (n=49)	1.53	6.12	1.59	6.25	*0.0067	*0.0022
SD	0.09	0.20	0.17	0.32		
7-9 (n=16)	1.54	5.96	1.59	6.10	0.1056	*0.0156
SD	0.08	0.22	0.16	0.26		
10-12 (n=2)	1.57	6.07	1.59	5.90		
SD	0.00	0.00	0.18	0.27	-	-
13-15 (n=10)	1.43	5.70	1.59	5.91	*0.0015	*0.0070
SD	0.18	0.32	0.17	0.27		
16-18 (n =3)	1.46	5.67	1.58	5.84	-	-
SD	0.18	0.30	0.14	0.27		
19-21 (n=3)	1.48	5.68	1.55	5.74	-	-
SD	0.19	0.32	0.12	0.19		
22-24 (n=6)	1.48	5.68	1.57	5.71	0.0974	0.3887
SD	0.12	0.30	0.17	0.26		

* Note: Student’s t-test, significance level of (5%).
